# Welfare Assessment on Pasture: A Review on Animal-Based Measures for Ruminants

**DOI:** 10.3390/ani10040609

**Published:** 2020-04-02

**Authors:** Chiara Spigarelli, Anna Zuliani, Monica Battini, Silvana Mattiello, Stefano Bovolenta

**Affiliations:** 1 Department of Agricultural, Food, Environmental and Animal Sciences, University of Udine, 33100 Udine, Italy; spigarelli.chiara@spes.uniud.it (C.S.); stefano.bovolenta@uniud.it (S.B.); 2 Department of Agricultural and Environmental Sciences-Production, Landscape, Agroenergy, University of Milan, 20133 Milano, Italy; monica.battini@unimi.it (M.B.); silvana.mattiello@unimi.it (S.M.)

**Keywords:** animal welfare, indicator, extensive, outdoor, cattle, sheep, goats

## Abstract

**Simple Summary:**

Welfare assessment in outdoor and extensive systems has rarely been investigated, and little is known about the most appropriate indicators. This study aimed at compiling a list of animal-based measures of welfare for domestic ruminants raised on outdoor/extensive systems by means of a systematic review. Out of 810 papers retrieved, 52 matched the inclusion criteria and went through an in-depth analysis. According to available literature, 45 indicators have been used to assess welfare on pasture, often following different methodologies. Most indicators were measured by observers even if the use of sensor technologies increased in recent years. Considering the growing interest in pasture-based or grass-fed products, it is suggested that welfare assessment in outdoor/extensive farming systems is carried out by following shared methodologies in order to provide evidence of the higher animal welfare claims that these products often imply compared to indoor systems.

**Abstract:**

Outdoor and extensive farming systems allow animals to behave in a natural way and are often perceived as welfare friendly. Nonetheless, the natural environment poses multiple challenges to the welfare of animals, sometimes hampering their capacity to cope. Welfare assessment in outdoor and extensive systems has been rarely investigated, and little is known about the most appropriate indicators. The aim of this review was to identify animal-based measures of welfare to apply in extensive and pasture-based systems in domestic ruminants. Through the use of a dedicated software for systematic reviews, 810 papers were screened and a total of 52 papers were retained for in-depth analysis. ABM resulting from these papers were initially divided according to the species (cattle and small ruminants, including sheep and goats) and then to four principles: comfort, behavior, feeding and health. The results showed that welfare data were collected applying different methodologies, with an increasing use of sensors in recent years. The need to herd and restrain animals for individual data collection is one of the major constraints to data collection in extensive farming systems. It is suggested that welfare assessment in outdoor/extensive farming systems is carried out by following shared procedures in order to provide evidence of the higher animal welfare claims that these products often imply compared to indoor systems.

## 1. Introduction

In the past half-century, animal production systems underwent a radical transformation that led to the concentration of large herds in fewer specialized intensive farms, where animals are usually kept indoors. This transformation and ultimately intensification of animal production [1] fueled a public debate on farm animal welfare and humane animal treatment. In response to the consumers’ growing concerns, several indicators and assessment methods were developed to allow a scientific measurement of welfare targeting indoor farming systems. Since animal welfare is a multidimensional concept [2], its proper assessment relies on the identification of complementary measures covering all dimensions [3]. The quality of the environment (e.g., bedding practices) or resources (e.g., water troughs) made available to the animal assessed with resource- and management-based (RBMs and MBMs) measures are considered as indirect indicators of animal welfare. Instead, direct indicators, or animal-based measures (ABMs), assess the response of an animal to the available resources and management practices. Recently, the importance of performing dairy cattle welfare assessment using ABM and acknowledging context-based variability in welfare outcomes was emphasized by the World Animal Health Organization [4] and the International Organization for Standardization [5]. The adoption of ABMs over non-ABMs is also encouraged by the European Food Safety Authority [6].

In Europe, the Welfare Quality^®^ (WQ) project [7] was one of the most important efforts towards the development of on-farm welfare assessment protocols compiling both ABMs and non-ABMs. The scores obtained are then collated to assess unit compliance with four main welfare principles (good feeding, good housing, good health and appropriate behavior). Finally, these principle scores are used to conclude on an overall evaluation.

Results on welfare assessment carried out with the above-mentioned methodologies highlighted that intensive housing systems could be associated with many behavioral and welfare problems [8], in contrast to pasture-based systems, which seem to be advantageous for animal welfare [9]. For example, many studies have suggested that pasture is beneficial for cows’ welfare because it leads to the reduction of hock damage, lameness and claw disorders [10,11,12]. Furthermore, grazing implies more moving activity, that can induce positive modifications of the animal’s metabolism, such as a more efficient clearance of plasma triacylglycerol’s, and this may have a positive effect on animals’ health and longevity [13]. In addition, outdoor and extensive farming systems allow animals to behave in a more natural way and due to all these reasons, they are often perceived as welfare friendly. Nonetheless, the natural environment poses multiple challenges to the welfare of animals (e.g., parasites, variable climate or predation), sometimes hampering their capacity to cope. Therefore, extensive farming systems may also cause poor welfare conditions if not properly managed [14,15]. In spite of this, welfare assessment in these systems has been investigated less frequently than in intensive rearing systems, and no official assessment method has been identified for these systems, despite the growing demand for pasture-based products [16].

This study aims at carrying out a review on animal-based measures of ruminants’ welfare in outdoor/extensive systems, in order to map the current available knowledge on the topic and compile an exhaustive list of established indicators for ruminants in outdoor/extensive systems that can be applied for welfare evaluation on pasture.

## 2. Materials and Methods 

A pre-defined protocol was established using the EFSA Guidance document on the application of systematic review methodology [17], which was developed considering the Cochrane Handbook [18] and according to the PRISMA (Preferred Reporting Items for Systematic Reviews and Meta-Analyses) statement [19]. A search of electronic databases (Scopus, Web of Science and PubMed) was carried out regarding ruminants’ welfare assessments in extensive and pasture-based systems and focused on scientific literature published from 1980 to 2019 using the following string: cattle OR cow* OR sheep OR goat* OR ruminant* AND assess* OR indicator* AND pasture OR outdoor OR extensive OR graz* AND evaluation OR measure* OR animal-based.

The search strategy of the review was defined according to the population (P) and outcome (O) format: Population: domestic ruminants (adult cattle (no calves), sheep and goats (no lambs, no kids), excluding buffalos); Outcome: animal-based measures of welfare assessed in pasture-based/extensive systems. 

The articles retrieved from the above-mentioned electronic databases had to meet the following criteria: (i) written in English; (ii) including only primary research; (iii) including animal-based welfare indicators measured on pasture-based/extensive systems. All direct indicators of welfare that can be recorded either by assessors looking at the animal, or by using sensors, were considered as animal-based measures, whereas indicators deriving from the laboratory analysis of biological samples (e.g., blood, milk, etc.) collected from the animals were excluded. 

Distiller SR (Distiller (Ottawa, Ontario), an online software for systematic reviews, was used to manage study selection and data extraction by two independent reviewers. At first, results from different databases were merged, and duplicates were removed. Study selection followed two steps: initial screening of titles and abstracts answering the question “Is the paper describing animal-based indicators of welfare for ruminants on extensive/pasture-based systems?”. Discrepancies were resolved by discussion and papers in full agreement or for which content was unclear were considered for screening of full text, while studies not answering the above-mentioned question were removed from the analysis. The second screening involved the full text examination and the description of each indicator considered in the study under review. Selected data were extracted and summarized in structured tables containing all assessments, the animal-based measures, their evaluation approach (by direct assessment (DA), video and/or audio recording (R), and/or sensor (S)), and the geographic location of the study. Divergences between reviewers were resolved by consensus or by a third reviewer, if necessary. The authors of the selected articles were not contacted for clarifications on missing or ambiguous data. 

## 3. Results and Discussion

**Figure 1:**
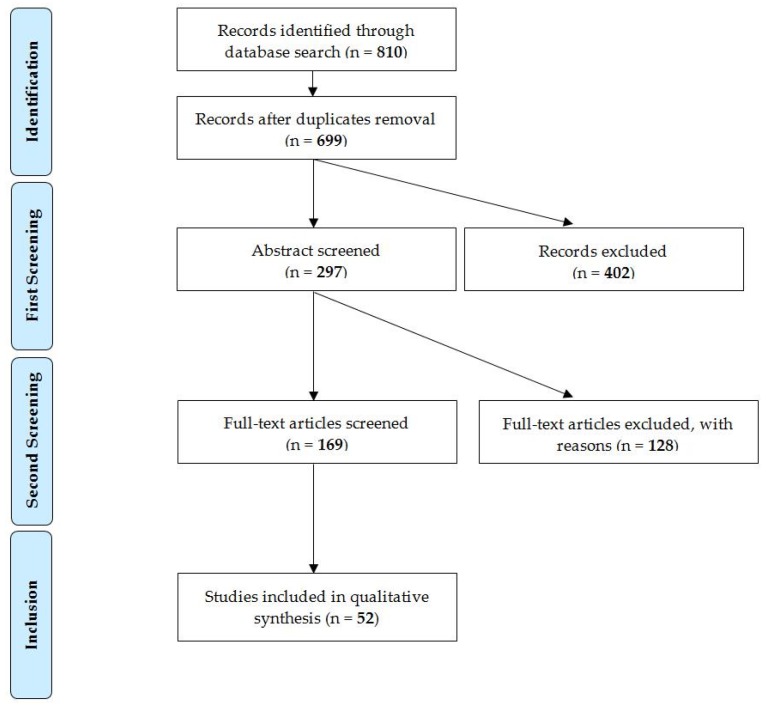
Flow chart of the systematic literature review process displaying exclusion and inclusion steps.

A total of 810 articles were recovered from the search of electronic databases following the above-mentioned inclusion criteria. Following the removal of duplicates, 699 articles were retained for first screening. In the next step, 169 articles were considered for full-text reading and 52 papers (i.e., 38 on cattle and 14 on small ruminants) matched all the inclusion criteria (Figure 1). 

**Figure 2:**
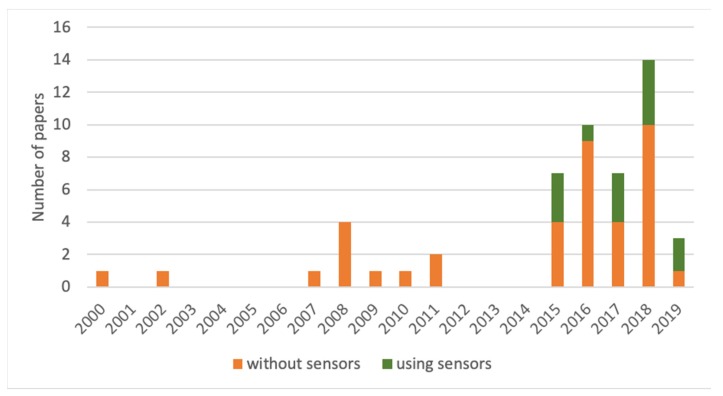
Total number of papers (involving or not involving the use of sensors) that met the inclusion criteria from 2000 to 2019 (no paper was retrieved from 1980 to 1999).

Despite the large number of papers retrieved at first screening, several were excluded from the analysis because they were assessing welfare before and/or after outdoor access [20,21], or because they were based on the collection of biological samples such as hair [22], blood [23], milk [24] and feces [25] and thus required the use of analytical methods to define the welfare status of animals on pasture. While such ABMs also allow the collection of relevant information on animal welfare on pasture, they were not, strictly speaking, measured on pasture. This point was considered as a way to check the actual feasibility of each measure on pasture and to ensure the relevance of the results produced through the systematic review. For what concerns the timeframe, in spite of the fact that the search period spanned almost 40 years (i.e., 1980-2019), papers meeting the inclusion criteria were published only between 2000 and 2019, with a remarkable increase in number after 2015 (Figure 2). This may be due to the fact that outdoor/extensive farming systems were of limited interest for animal welfare scientists until recent years. In this regard, even if ABMs such as body condition were collected in early years by animal scientists, they would be described as production performance parameters using terminology that did not match our search string. It is interesting that only 25% of the studies reported in the selected papers involved the use of sensors, with a trend to increase this use in the last years, starting from 2015 (Figure 2).

The indicators extracted were assigned to four principles, inspired by WQ® classification: comfort, behavior, feeding and health. The results are presented separately for cattle (including both dairy and beef cattle), and for small ruminants (sheep and goats) and separate tables were compiled for each criterion. For cattle, the production type was also specified (dairy or beef), while for small ruminants only the species (sheep or goats) was described, considering that small ruminants at pasture are mostly viewed as dual purpose animals, and therefore it was difficult to assign them to a specific production type.

### 3.1. Animal-Based Measures for Cattle on Extensive/Pasture-Based Systems

We identified 33 animal-based measures for cattle (Table 1, Table 2, Table 3 and Table 4). 

Table 1 displays seven ABMs concerning the comfort principle, reported in 25 papers deriving from studies carried out in all continents, and the evaluations were mainly carried out on dairy cows and by direct assessment. Most of authors evaluated cleanliness as yes–no binary rating, while only two [26,27] preferred to consider the animal score on a four- or five-point rating scale from clean to dirty. Hernandez et al. [31] were the only authors evaluating animals at the milking parlor during milking all the others did it at pasture. Animal position on pasture (lying, resting, sitting or standing) was frequently assessed. Direct assessments mainly considered the time spent resting on the ground [39] or standing still [40], while authors who used sensors such as pedometers, mostly monitored the number of lying bouts and their duration [36]. The use of sensors may be related to the difficulty of individually measuring these indicators. Time spent lying can be an indicator of welfare issues, for example lying was identified by Thompson et al. [33] as an effective indicator of lameness in grazing systems, but the effect differs depending on both the severity of lameness and the type of lying surface. On the other hand, several authors [32,36] found a positive influence of grazing and comfortable surfaces on lying movements and duration. Standing [36] and standing still with the head raised [45,46] were identified as a potential warning signal for inadequate feed allocation. Concerning rising movement [44], the indicator is of limited importance on pasture condition as it aims at assessing the adequacy of available farm structures, even if longer rising times may be linked to feet injuries and locomotion issues similar to what was found for lying movements and duration. However, unless recorded with sensors, such indicators are extremely time consuming to collect and may be prone to observers’ bias, reducing the feasibility of such indicators for welfare assessment on the pasture. Concerning sitting behavior [41], it seems a rare finding on pasture and may describe a prolonged response to poor availability of on-farm resources. It is thus not considered a relevant ABM, at least for year-long grazing animals. 

The use of shade or shelter was assessed as the passage of the animals to and from the water source or sun protection. Despite the great importance of shade at pasture for ensuring thermal comfort, few authors [24,39,48,49] considered this indicator, probably because the number of trees is usually considered as a resource-based and not as an animal-based measure. Nonetheless, when access to shade was provided, cows spent less time at the water trough and laying down, and chose to perform behavioral activities, including grazing, in the shade emphasizing the benefits of silvo-pastoral systems for animal welfare.

Table 2 summarizes the ABMs found in 21 papers related to the behavior principle to be collected in extensive conditions. From these papers, we identified 11 ABMs. Behavior principle is, indeed, characterized by a wide diversity of application, including daily activities, social interactions, human–animal relationships, and the assessment of emotional state. Most ABMs (68.85%) are recorded by direct assessment, followed by video-recording (22.95%, that also include vocalizations collected by sound recording), and sensors (in only 8.20% of cases). The use of sensors was only limited to those papers that investigated activities such as walking (e.g., [34,37,47]) and consists of data loggers attached to the hind legs or neck of the animals. Pedometers are not expensive and are already commonly used in many farms to record heat or to allow animals to be milked by automatic systems. Their use in extensive husbandry systems can provide information on the spatial behavior of cattle. However, more expensive sensors may be of use to investigate behaviors other than walking: spatial proximity loggers collect data on associations between cows and allow us to gather information on social networks and affiliative behaviors [53]. Cost may be a limit on the use of these sensors, but they can provide detailed information on the relationships and changes in behavior of the herd during the year. 

Most behaviors are collected by direct assessment. Direct assessment can be adopted for behavioral observations and for indicators that require a test performed by humans, as in the case of the evaluation of human–animal relationships using an avoidance distance test [29,30,50]. These authors did not report any feasibility constraint; however, according to Hernandez et al. [31], approaching animals in extensive systems may be difficult and sometimes not very informative as cattle bred in large groups in extensive systems may avoid the human touch, even if not necessarily afraid of it. The feasibility of direct assessment for behavioral observations is often low, especially in extensive/pasture-based systems: observations are usually time consuming (e.g., [41] up to 24 h/day), many assessors need to be trained (e.g., [42] trained six observers), and, furthermore, information provided about inter-observer reliability is not always sufficient ([32] tested the inter-observer reliability of three trained assessors before applying the welfare protocol). The method most frequently used to record behaviors is the instantaneous and scan sampling method [38,41,42].

Direct assessment was also used to assess animal emotions and the only indicator identified to this aim is Qualitative Behavior Assessment (QBA). Some authors [30,32] reported more positive emotional states of cattle at pasture compared to animals kept indoors. Although QBA received some criticisms, mainly due to possible bias in judgment [54] or subjectivity [31], it is important to notice that, when performing direct observations, observers are always unavoidably aware of the type of husbandry systems they are assessing, and this may concern both quantitative and qualitative indicators [54], thus affecting their perception. However, a study conducted on dairy goats kept in indoor and pasture-based systems reported that if assessors receive an effective QBA training, this can help in overcoming the influence of an environment perceived as more “welfare friendly” [55]. The feasibility of QBA in extensive systems is high as observations last at most 20 minutes, followed by few minutes where the assessor scores the descriptors. Some situations may require the use of binoculars in order to observe the animals at a distance and avoid disturbing their activities. Video-recording for behavioral observations were mainly used to record social behaviors as cohesive and agonistic behaviors. The time of recording, when provided, is relatively limited ([31] recorded the animals at pasture for only two hours) and sometimes influenced by factors, e.g., weather, temperature, routine changes, and animal behavior. Although the use of video-recording may increase the feasibility of an indicator, further research is needed in order to gather information on the right time for recording, including the best moment of the day to register a specific behavior and the sufficient length of the recording.

Some papers included indicators already tested for indoor husbandry systems and the authors stated that they selected the most feasible indicators for extensive systems. However, valid and feasible indicators for indoor systems need to be tested again and sometimes adapted to be used in extensive systems. In most cases, insufficient information is provided about selection criteria or other useful information that can be extrapolated to suggest the use of a specific indicator for pasture-based systems.

Table 3 shows a total of six ABMs concerning the feeding principle, and 26 scientific papers investigating a link between these measures and animal welfare. The measurements were mainly carried out by direct assessment, while in only a few cases were sensors used. Sixty-nine per cent of the measures concerned dairy cows and the remaining 31% concerned beef cows. Latin America is the geographic area where most of the experiments were carried out. 

A measure widely used to evaluate the nutritional status of animals, in particular dairy cows, refers to the amount of stored body fat. The body condition score (BCS) method [61] allows us to estimate the general body fat by means of a visual (or, less frequently, tactile) evaluation of the quantity of subcutaneous fat in certain body regions of the animal (essentially the tail head cavity, pin bones, rump, short ribs, backbone). In contrast to the measure of body weight, BCS is not affected by body size, by intestinal filling or by pregnancy status. The lowest value of the BCS indicates a very lean condition (linked to a serious underfeeding and/or a disease state), while the highest value indicates a very fat condition (linked to an overfeeding and consequent risk of metabolic diseases). Monitoring the BCS of grazing dairy cows is extremely useful and allows us to evaluate the energy balance in the various phases of the lactation cycle. Long periods on pasture with low energy intake cause an energy deficiency responsible for alterations in milk composition, milk yield and lactation persistency [62], and may be also related to reproductive performance [63]. During the grazing period, it is not always easy to fulfill dairy cows’ nutritional requirements only through grazing. The BCS therefore allows the breeder to understand if there is a need for food supplements in order to avoid hunger and nutritional imbalances. 

In the selected papers, several types of scores were chosen to assess the BCS as a welfare indicator of grazing animals. For dairy cows, in experiments conducted in Italy and Mexico, a score of 0–2 was used, in line with the WQ assessment protocol for cattle [28,29,31,44,56], while in other countries and situations a score of 1–5 [27,33,35,57,58] or 1–10 [59] was used. Other authors [30] used a score of 1–9 for grazing beef cows. The review did not identify experiments that used 3D cameras to monitor the BCS of cattle in extensive situations, which may represent a promising and time-saving assessment option in the future [64], considering the importance of body condition assessment on pasture.

In extensive systems, particular attention must be paid to water provision. Authors evaluated water utilization by using different methods: the time spent drinking [41,45,48], the percentage and number of animals moving to water sources [31,42], rather than the access (free or limited) to the source [57]. Some authors analyzed the consumption of water, through the presence of signs of dehydration on the animal [30] or by indicating the urinating actions [45]. Water provision and cow’s welfare are closely connected, and climate change might further compromise animal well-being especially during the second phase of the grass vegetative stage or in geographical areas affected by droughts. Lardner et al. [65] and Coimbra et al. [66] underline the link between drinking behavior and body size, dry matter intake, production stage, air and water temperature, quality or type of water access. Thus, if not contextualized, the estimated daily average intake per animal at the troughs provides limited information on water requirement. On the other hand, a sign of dehydration seems a rather demanding measure to be taken in pasture-based and extensive systems, limiting the potential role of ABMs in the assessment of adequate water provision.

The evaluation of the feeding behavior of grazing cattle, in place of or in addition to the BCS, allows us to respond adequately to the feed requirements in terms of animal welfare. The availability of data regarding the feeding behavior of grazing cows allows the breeder to identify specific individual problems and act to restore the best conditions for animal welfare. In the past, these measurements were mainly carried out using visual methods (e.g., Tucker et al. [39] with instantaneous scan sampling) and still today many authors, such as those identified in this review, adopt these rather than analytical methods which are more time consuming (e.g., Bovolenta and colleagues [25,67], estimating herbage intake using the n-alkane method). Grazing and rumination is positively related to feeding time and dry matter intake. Following periods of high feed intake, cows spend more time ruminating, usually after a 4-h lag. In recent years, the tools of "precision livestock farming" [68], adopted and developed indoors in order to optimize the use of resources and improve the productive and reproductive performance of animals, have also been proposed for the pasture environment [69], and could represent a radical change in terms of the feasibility and effectiveness of animal welfare monitoring in extensive systems. Some selected papers [26,46,47,48,60] have proposed electronic equipment (in particular behavior-monitoring collars, GPS devices, pedometers) for the continuous monitoring of feeding and locomotion behavior, which has proven to be efficient and reliable. 

Table 4 displays 12 animal-based measures related to the health principle of large ruminants on pasture. Most indicators were measured by assessors through the direct observation of dairy cattle. While some measures were well-established indicators of health in indoor intensive systems and followed the WQ assessment methodology [74], others were specifically developed for grazing animals. For example, hoof and leg injuries, as well as integument and body alterations, represent major welfare issues for housed cattle and are among the most important reasons for culling. In particular, an open shoulder is an indicator of reduced tonicity, mostly found in pluriparous cows housed in permanent tie-stall systems and it may be an indicator of limited importance in year-round pasture-based systems. The pasture is also considered to be a protective factor against claw disorders and lameness [12,75] according to several studies that compared the occurrence of such conditions between indoor and pasture-based systems [28,30]. Nonetheless, claw disorders and lameness do also represent a significant welfare issue in pasture-based systems, and thus should be constantly monitored. Despite no studies identified through this systematic review reporting the use of sensors, smart technologies could also play a role in the early detection of claw and locomotion disorders in grazing animals. Natural environments could also represent a risk for health and pose challenges for grazing animals. For example, diet composition cannot always be controlled in extensive systems and improper forage intake may result in gastrointestinal disorders. Signs of diarrhea, softer feces and bloated rumen were the indicators of gastrointestinal disorders assessed in dairy [44] and beef [30] cattle. Pasture access may also increase the risk of both endo- and ectoparasite infestation. While signs of endoparasite infestation may be assessed through body condition measurement or the observation of gastrointestinal disorders, the presence of ectoparasites was assessed through direct observation of parasites on hides or through the effects of their infestation such as skin lesions or ocular discharges [29,30]. Exposure to climate variability and extreme weather (e.g., heat waves) are a further challenge for grazing animals. Assessment of thermal stress was performed by observing respiration patterns or through temperature measurement. Unless recorded with laser thermometers as described by Morales and colleagues [30], the measurement of body temperature appeared not suitable for beef cattle systems in which chances for animal restrain are little compared to dairy systems. In this regard, the direct observation of respiration patterns and rates may represent a better choice for all systems and production types, until new technologies will allow the remote monitoring and recording of body temperature, effectively combining the early detection of heat imbalances and disease occurrence.

### 3.2. Animal-Based Measures for Small Ruminants on Extensive/Pasture-Based Systems 

For small ruminants, 20 ABMs were extracted from 14 studies carried out in Australia, the UK and, to a lesser extent, in Italy, France, and Argentina (Table 5, Table 6, Table 7 and Table 8). Most of the studies (86%) were carried out on sheep, only one focused exclusively on goats [55], and one paper dealt with both species [84]. This is probably due to the higher economic importance of sheep and to their management system, which is almost exclusively pasture-based, whereas goats are often raised in intensive or semi-intensive systems, especially in more developed countries. In most cases (71% of the articles), all the indicators were collected by direct assessment, whereas sensors were used for data collection in 21% of the studies, and in one study [80], both approaches were adopted. The use of sensors based on omnidirectional accelerometers [80,81,83] was helpful for the assessment of activities related to comfort, behavior and feeding principles, and the integration with GPS devices [81] provided additional interesting and detailed results on spatial behavior and movements (that could be associated with feeding behavior), even in a very extensive context, without disturbing the animals. This is obviously much less time-consuming than carrying out direct or video-recorded observations, whose feasibility on farms can be considered quite low, due to the long observation time required to detect irregularities in behavioral rhythm that may be indicative of health and welfare issues. However, McLennan et al. [80] suggest that the level of detail provided by accelerometer devices needs to be further improved, as in their study, high levels of accuracy could only be obtained for gross behavior categories (low vs. medium/high activity level).

It also has to be noticed that both [80,81] present interesting methodological approaches for the collection of behavioral data using sensors, and mention the importance of monitoring behavior as a good indicator of animal welfare, but they do not provide clear indications as to how to interpret the results. Therefore, the validity of behaviors such as walking, grazing or searching for food as indicators of animal welfare has not been discussed in these studies. Within the behavior principle, the results of [83] on the assessment of circadian rhythms of general activity using the Degree of Functional Coupling (DFC, which expresses the percentage of the measured behavior that is harmonically synchronized with environmental rhythms, over a 24-h period) provide reliable information on sheep welfare: high DFCs indicate high synchronization, which is considered a positive indicator of animal welfare [89].

Another interesting measure related to the behavior principle was used by Munoz et al. [82] to investigate the quality of human–animal relationships: the ewe’s response (flight distance and behavior reaction) to an unfamiliar human was evaluated in a small random sample of sheep in a holding pen. The execution of the test in the pen can be feasible; however, its validity and reliability under this specific situation have not been investigated. 

As to the feeding principle, another promising application of sensors is described by the study of Gonzalez-Garcia et al. [88], who used a remote weighing prototype based on the walk-over-weighing concept, combined with radio-frequency identification, that allowed them to record sheep body weight in extensive conditions, with no need to restrain the animals. The direct assessment of body weight was carried out by McGregor et al. [84]: these authors could not confirm the importance of live weight as a welfare indicator, but highlighted the importance of BCS, which was significantly correlated with mortality rate in Angora goats. Although not described in detail in this paper, both body weight and BCS probably implied restraining the individual animals, and were therefore time-consuming. The same time constraints apply to body condition scoring carried out by other authors [76,77,82,85,86,87]. 

Furthermore, for other ABMs, such as cleanliness [76,77,79,82], or health indicators (e.g., integument alterations, fleece conditions, or foot lesions [76,77,79,82]), the evaluation was carried out by assessors, and the animals had to be restrained in small holding pens to allow individual examination; for the evaluation of mastitis, restraining the animals in a crate was also required [82]. These operations were therefore time-consuming and probably induced some level of stress in animals that were not used to being handled due their extensive living conditions. In the case of Munoz et al. [79], it is worth noticing that the selection of the individual animals to be inspected was grounded on an appropriate sampling scheme based on a power calculation assuming a 50% prevalence of the trait under observation. The selection of appropriate sampling schemes is very important, especially when dealing with large herds (as sheep often are) and when animals have to be herded for the inspection, which is a common situation in extensive farming systems. Angell et al. [76,77] also included the evaluation of lameness, that was scored by a trained assessor in a holding pen, while Munoz et al. [79,82] used a similar locomotion score but evaluated it when the sheep were released from the holding pen. 

Phythian et al. [78] used a different approach for lameness evaluation in sheep, that did not require to herd the animals: a group-level assessment was performed by an assessor who briefly observed the flock at a distance for five minutes, and then counted the number of lame animals based on the observation of behavioral cues (e.g., nodding of head, grazing on knees, uneven gait, etc.), rather than assigning a lameness score as in Angell et al. [76,77]. Phythian et al. [78] adopted the same practical approach for recording other ABMs: coughing, breech soiling, abdominal soiling, pruritis, wool loss, and “dull physical demeanour”. Additionally, these authors applied a Qualitative Behavior Assessment, which only required an average time of 30 min/farm for flocks of up to 120 sheep, observed from a distance with no need to enter the field. Interestingly, some QBA descriptors were correlated with other welfare measures (e.g., the proportion of lame sheep and of sheep with “dull physical demeanour” was correlated with descriptors like distressed, dull and dejected), providing evidence of the concurrent validity of these measures. QBA was also applied on goats, using a similar feasible procedure, and highlighted interesting differences between the emotional state of goats on pasture vs. indoor housing, with a good inter-observer reliability [55].

Additional information about the reliability of ABMs for small ruminant welfare assessment is provided by Munoz et al. [79], who found poor agreement for rumen fill, foot-wall integrity, and hoof overgrowth, and considered fleece cleanliness not be meaningful for extensive systems. Based on these considerations, the authors suggest the use of body condition score, fleece condition (based on lumpiness or signs of ectoparasites), skin lesions, tail length, dag score and lameness for on-farm welfare assessments of extensive managed sheep, as all these measures are also feasible due to the fact that they do not require any specialized equipment. Tail length was listed as an ABM [79,82] despite the fact that it may be considered as a risk factor for several conditions such as rectal prolapse, flystrike and bacterial arthritis. Furthermore, Munoz et al. [79] consider that most of these measures (e.g., thin body condition, lameness and dag score) can be visually recorded from a distance viewing sheep in their paddock, rather than in holding pens, with minimal interference with farm work. This suggestion is supported by the successful collection of similar measures by Phythian et al. [78], as reported above. Furthermore, Munoz et al. [79] suggest that the lactation period may not be the best time to carry out the evaluation due to the presence of lambs.

## 4. Conclusions

This study aimed at compiling a list ABMs of welfare for domestic ruminants raised on outdoor/extensive systems by means of a systematic review. The results showed that welfare data were often collected applying different methodologies. Considering the growing interest in pasture-based or grass-fed products, and not neglecting the role of suitable structures or management, it is suggested that welfare assessment in outdoor/extensive farming systems is carried out with selected ABMs following shared approaches, to provide evidence for the higher animal welfare claims that these products often imply. In addition, the use of sensors has become more and more common in recent years. The development of these tools is a very promising opportunity to record welfare measures in extensive/pasture-based systems, where it is often difficult to have direct and close access to the animals, and where the collection of individual records might require time-consuming and potentially stressful operations, such as herding and restraining. It is probably not a coincidence that the number of these studies has increased since 2015, when the use of sensors became more common. Furthermore, sensors do not require the presence of an observer, which can bias the results of the assessment. It is expected that in the future, the tools of "precision livestock farming" adopted and developed for indoor systems will be extensively applied to pasture-based systems in order to further improve the productive and reproductive performance of animals, together with their health and welfare. 

## Figures and Tables

**Table 1:** Animal-based measures (ABMs) evaluated on cattle concerning the comfort principle.

ABMs	Assessment	Unit	Production Type	Evaluation Approach ^1^	Country	Ref.
Cleanliness	plaques of dirt on legs and udder	score 1–5	beef	DA	IRL	[26]
		score 1–4	dairy	DA	IND	[27]
		yes/no	dairy, beef	DA	ITA, MEX	[28,29]
	hind legs score and ventral part score	yes/no	beef	DA	COL	[30]
	degree of dirt on the body parts	yes/no	dairy	DA	MEX	[31]
Lying	duration of lying	seconds	dairy	DA	MEX, DEU	[31,32]
		min/bout	dairy	S	BRA	[33]
		min/day	dairy	S	USA, IRL	[34,35,36]
	number of lying bouts	bouts/day	dairy	S	BRA, USA, IRL	[33,34,35,36]
			beef	R	IRL	[26]
				S	AUS	[37]
		frequency of events	beef	DA	MEX	[38]
	lying still	min/day	dairy	S	USA, IRL	[34,35,36]
		hours/day	dairy	S	BRA	[33]
				DA	NZL	[39]
			beef	R	IRL	[26]
		% of time	dairy	DA	BRA	[40]
			beef	DA	FIN	[41]
				S	AUS	[37]
		% animals	dairy	DA	DEU	[32]
			beef	DA	URY, MEX	[42,43]
	hampered lying down movements	% events	dairy	DA	ITA	[44]
Resting	maintained standing or lying position	% of time	beef	DA	JPN	[45]
			dairy	DA + S	GBR	[46]
Sitting	abnormal posture with forelimbs extended	% of time	beef	DA	FIN	[41]
Standing	standing still	% of time	beef	S	AUS	[37]
				DA	FIN	[41]
			dairy	DA	BRA	[40]
		min/day	dairy	S	ITA	[47]
		hours/day	beef	R	IRL	[26]
			dairy	DA	NZL	[39]
		% of animals	beef	DA	URY	[42]
Rising	incorrect rising events, duration	% events, seconds	dairy	DA	ITA	[44]
Use of shade/shelter	time spent in shade	hours/day	dairy	DA	NZL	[39]
		% of time	dairy	S	BRA	[48]
				R	BEL	[24]
	time spent in natural and artificial shelter	% of time	beef	S	BEL	[49]

^1^ direct assessment: DA; recording (audio and/or video): R; sensor: S.

**Table 2:** Animal-based measures (ABMs) evaluated on cattle concerning the behavior principle.

ABMs	Assessment	Unit	Production Type	Evaluation Approach ^1^	Country	Ref.
Vocalization	animals vocalizing	number of animals	beef	R	MEX	[43]
Qualitative behavior assessment	descriptors on a VAS scale	0–125 mm	dairy	DA	DEU, MEX	[31,32]
			beef	DA	COL	[30]
Avoidance distance test	flight distance	0–200 cm	dairy	DA	ITA, MEX	[31,44]
			beef	DA	COL, MEX	[29,30]
		0–300 cm	dairy	DA	ITA	[50]
Behavior during restraint	behavior (very calm- struggling)	score 1–5	beef	DA	BRA	[23]
Entry and exit speed	speed(walk-run)	score 1–3	beef	DA	BRA	[23]
Stereotypy	tongue-rolling	% of time	beef	DA	FIN	[41]
		% of events	dairy	DA	ITA	[44]
	bar-biting	% of time	beef	DA	FIN	[41]
	water lapping	% of events	dairy	DA	ITA	[44]
	licking objects	% of animals	beef	DA	URY	[42]
Comfort behavior	self-grooming	% of animals	beef	DA	URY	[42]
		% of time	beef	DA	FIN	[41]
		frequency, seconds	beef	DA	JPN	[45]
	grooming with trees	frequency, seconds	beef	DA	JPN	[45]
Cohesive behavior	allo-grooming	frequency, seconds	beef	DA	JPN	[45]
		frequency of events	dairy	DA + R	MEX	[51]
				R	MEX	[31]
				DA	CAN	[52]
			beef	DA	COL	[30]
		% of observations	beef	DA	FIN	[41]
		animals involved	dairy	DA	CAN	[52]
		duration (min/animal)				
	playful horning	frequency of events	beef	DA	COL	[30]
		number of events	dairy	R	MEX	[31]
Explorative behavior	chewing objects (licking, gnawing, masticating)	% of time	beef	DA	FIN	[41]
Agonistic behavior	head-butts	frequency of events	dairy	R	MEX	[31]
				DA + R	MEX	[51]
			beef	DA	FIN	[41]
			beef	DA	COL	[30]
			dairy	DA	DEU	[32]
			beef	R	MEX	[29]
	feints	frequency of events	beef	DA	FIN	[41]
	displacements	frequency of events	dairy	DA	DEU	[32]
			beef	DA	COL	[30]
				R	MEX	[29]
			dairy	R	MEX	[31]
		% of time	dairy	R	BRA	[48]
	chases	frequency of events	beef	DA	COL	[30]
			dairy	R	MEX	[31]
	fights	frequency of events	beef	DA	COL	[30]
			dairy	R	MEX	[31]
	standing animals towards a standing counterpart	frequency of events	beef	R	IRL	[26]
Other activities	standing idleness lying idleness	% of time	dairy	R	BRA	[48]
			beef	DA	MEX	[38]
			dairy	DA	BRA	[40]
	walking without grazing	% of time	dairy	DA	BRA	[40]
			beef	DA	MEX, FIN	[38,41]
		min/day	dairy	S	ITA	[47]
		number of steps	beef	S	AUS	[37]
			dairy	S	USA, ITA	[34,47]
		number of animals	beef	DA	JPN	[45]
		% of animals	beef	DA	URY	[42]
		% of time	dairy	DA + S	GBR	[46]
	cow-calf proximity	distance (m)	beef	DA	MEX	[43]

^1^ direct assessment: DA; recording (audio and/or video): R; sensor: S.

**Table 3:** Animal-based measures (ABMs) evaluated on cattle concerning the feeding principle.

ABMs	Assessment	Unit	Production Type	Evaluation Approach ^1^	Country	Ref.
Body condition	BCS ^2^	score 0–2	beef	DA	MEX	[29]
		score 0–2	dairy	DA	ITA, MEX	[28,31,44,56]
		score 1–5	dairy	DA	IRL, BRA, IND	[27,33,35,36,57,58]
		score 1–9	beef	DA	COL	[30]
		score 1–10	dairy	DA	NZL	[59]
Drinking	animals drinking and moving to water	% of animals	beef	DA	URY	[42]
	access to water source	number of animals	dairy	DA	MEX	[31]
		% of time	dairy	DA	BRA	[57]
	time spent drinking	% of time	beef	DA	FIN, JPN	[41,45]
			dairy	S	BRA	[48]
Sign of dehydration	skin elasticity and enophthalmia	yes/no	beef	DA	COL	[30]
Urinating ^3^	action	% of time	beef	DA	JPN	[45]
Eating	grazing and browsing	% of time	beef	DA	JPN, FIN, MEX	[38,41,45]
			dairy	DA + S	GBR	[46]
				S	BRA	[48]
				DA	BRA	[40]
		minutes and % of time	beef	DA + S	CAN	[60]
		hours/day	dairy	DA	NZL	[39]
				R	MEX	[51]
			beef	R	IRL	[26]
		frequency of events	dairy	DA	CAN	[52]
		% of animals	beef	DA	URY	[42]
	grazing time, grazing bites	min/day, number/day, number	dairy	S	ITA	[47]
	grazing intensity	bites/day	beef	R	IRL	[26]
			dairy	S	ITA	[47]
Rumination	ruminating (performing regurgitation and movements with the jaw)	% of time	beef	DA + S	CAN	[60]
				DA	JPN, FIN, MEX	[38,41,45]
			dairy	S	BRA	[48]
				DA	BRA	[40]
		min/day	dairy	S	ITA	[47]
			beef	DA + S	CAN	[60]
		% of animals	beef	DA	URY	[42]
	rumination bite, *bolus* (cud), rumination intensity	number/day, number/day, number bites/day or *bolus*	dairy	S	ITA	[47]

^1^ Direct assessment: DA; recording (audio and/or video): R; sensor: S. ^2^ BCS: subcutaneous fat stores based on visual evaluation of several body region. ^3^ Urinating, drinking, walking and grooming are recorded jointly as a single indicator.

**Table 4:** Animal-based measures (ABMs) evaluated on cattle concerning the health principle.

ABMs	Assessment	Unit	Production Type	Evaluation Approach ^1^	Country	Ref.
Lameness	lameness	yes/no	dairy	DA	MEX, ITA	[31,44]
			beef	DA	MEX	[29]
	severe lameness	yes/no	dairy	DA	ITA	[28]
	locomotion score	score 1–5	dairy	DA	IRL, USA, BRA, IND	[27,33,40,58,70,71]
		score 1–4	dairy	DA	AUS	[72]
		score 0–3	dairy	DA	NZL	[73]
	limping of any type	yes/no	beef	DA	COL	[30]
	spine curvature, tracking, adduction/abduction, speed and head bob	score 1–5	dairy	DA	IRL	[35]
Claw alterations	heel erosion and dermatitis	score 0–5	dairy	DA	IRL	[35]
	sole thickness	millimeters	dairy	S	USA	[71]
	claw overgrowth	yes/no	dairy	DA	ITA	[28,44]
		score 1–4	dairy	DA	IND	[27]
	hoof abnormalities	yes/no	dairy	DA	BRA	[58]
Integument alterations	hairless patches, lesions, swellings/inflammation	yes/no	beef	DA	COL	[30]
			dairy	DA	MEX, ITA	[28,31]
		number of cases	dairy	DA	ITA	[28,44]
			beef	DA	MEX	[29]
		score 1–4	dairy	DA	IND	[27]
Body alterations	open shoulder	yes/no	dairy	DA	ITA	[44]
Respiration	panting score (respiratory rate, deepness of panting, degree of drooling)	score 0–4.5	dairy	DA	BEL	[24]
	respiration rate (flank movements)	breaths/min	dairy	DA	BEL	[24]
	hampered respiration	yes/no	beef	DA	MEX, COL	[29,30]
			dairy	DA	MEX, ITA	[28,31]
Coughing and sneezing	coughs episodes	yes/no	dairy	DA	MEX, ITA	[28,31,44]
			beef	DA	COL	[30]
		number of episods/animal/15min	beef	DA	MEX	[29]
	sneezes episodes	number of episods/animal/15min	beef	DA	MEX	[29]
Discharges	vulvar discharge	score 1–4	dairy	DA	BRA	[57]
		yes/no	beef	DA	MEX	[29]
			dairy	DA	ITA	[28,44]
	ocular and nasal discharge	yes/no	beef	DA	MEX, COL	[29,30]
			dairy	DA	ITA, MEX	[29,31,44]
Diarrhea	diarrhea	yes/no	beef	DA	COL, MEX	[29,30]
			dairy	DA	MEX, ITA, IND	[27,28,31]
	soft feaces	yes/no	dairy	DA	ITA	[44]
Bloat rumen	Presence bloated rumen	yes/no	dairy	DA	MEX	[31]
Parasites	ectoparasites	yes/no	beef	DA	MEX, COL	[29,30]
Body temperature	skin temperature	C°	beef	S	COL	[30]
	vaginal temperature	C°	dairy	S	NZL	[39]
	rectal temperature	C°	dairy	S	BEL	[24]
			beef	S	IRL	[26]

^1^ Direct assessment: DA; recording (audio and/or video): R; sensor: S.

**Table 5:** Animal-based measures (ABMs) evaluated on small ruminants concerning the comfort principle.

ABMs	Assessment	Unit	Species	Evaluation Approach ^1^	Country	Ref.
Cleanliness	plaques of dirt on tail and perineal wool	score 0–3	sheep	DA	GBR	[76,77]
	soiling on breech and abdominal region	% of animals affected	sheep	DA	GBR	[78]
	fleece cleanliness	score 0–3	sheep	DA	AUS	[79]
Lying (excluding rumination while lying)	lying on ground with no jaw movement	% of time (total counts/min)	sheep	DA + S	GBR	[80]

^1^ direct assessment: DA; recording (audio and/or video): R; sensor: S.

**Table 6:** Animal-based measures (ABMs) evaluated on small ruminants concerning the behavior principle.

ABMs	Assessment	Unit	Species	Evaluation Approach ^1^	Country	Ref.
Qualitative behavior assessment	descriptors on a VAS scale	0–125 mm	goats	DA	ITA	[55]
			sheep	DA	GBR	[78]
Alert	vigilance episods	% of time	sheep	S	ARG	[81]
Human–animal relationship	flight distance	meters	sheep	DA	AUS	[82]
	behavior score (from calm to escape)	score 0–3	sheep	DA	AUS	[82]
Apathy (dull demeanour)	animal with lowered head carriage, showing behavioral separation from the rest	% of animals affected	sheep	DA	GBR	[78]
Walking	walking fast	% of time	sheep	S	ARG	[81]
	moving forward with the head up	% of time	sheep	DA + S	GBR	[80]
Circadian rhythms	% of harmonic/synchronized cyclic behavior	Degree of Functional Coupling	sheep	S	GBR	[83]

^1^ direct assessment: DA; recording (audio and/or video): R; sensor: S.

**Table 7:** Animal-based measures (ABMs) evaluated on small ruminants concerning the feeding principle.

ABMs	Assessment	Unit	Species	Evaluation Approach ^1^	Country	Ref.
Body condition	BCS ^2^	score 1–4	sheep + goats	DA	AUS	[84]
		score 1–5	sheep	DA	AUS, ITA, GBR	[76,77,79,82,85,86]
		score 0–5	sheep	DA	GBR	[87]
	body weight	kg	sheep	S	FRA	[88]
			sheep + goats	DA	AUS	[84]
Eating	grazing	% of time	sheep	DA + S	GBR	[80]
		% of time	sheep	S	ARG	[81]
Rumination	resting-rumination	% of time	sheep	S	ARG	[81]
	ruminating or regurgitating a bolus (standing or lying down)	% of time	sheep	DA + S	GBR	[80]
Searching food	searching for food	% of time	sheep	S	ARG	[81]
Rumen fill	evaluation of the animal’s left-hand side (sunk or convex)	yes/no	sheep	DA	AUS	[79]

^1^ direct assessment: DA; recording (audio and/or video): R; sensor: S. 2 BCS: subcutaneous fat stores based on visual evaluation of several body region.

**Table 8:** Animal-based measures (ABMs) evaluated on small ruminants concerning the health principle.

ABMs	Assessment	Unit	Species	Evaluation Approach ^1^	Country	Ref.
Lameness	nodding of head, grazing on knees, uneven gait during locomotion, difficult rising, affected limb when standing	% of animals affected	sheep	DA	GBR	[78]
	locomotion score	score 0–3	sheep	DA	GBR, AUS	[76,77,79,82]
Integument alterations	skin lesions	number, location and score 1–4	sheep	DA	AUS	[82]
Cough	paroxysmal coughing, respiratory distress, breathing and wheezing	% of animals affected	sheep	DA	GBR	[78]
Pruritis	rubbing or scratching against objects, restlessness, stamping of feet, biting and nibbling	% of animals affected	sheep	DA	GBR	[78]
Wool loss	areas of fleece loss	% of animals affected	sheep	DA	GBR	[78]
Fleece	fleece condition	score 0–2	sheep	DA	AUS	[79,82]
	dag score	score 0–5	sheep	DA	AUS	[79,82]
Mastitis	physical inspection of the udder (presence of fibrosis, swelling, inflammation, abscesses)	score 0–4	sheep	DA	AUS	[82]
Tail length	tip of the vulva covered by the tail	yes/no	sheep	DA	AUS	[79,82]
Claw alterations	foot-wall integrity	score 0–3	sheep	DA	AUS	[79]
	hoof overgrowth	score 0–2	sheep	DA	AUS	[79]
	contagious ovine digital dermatitis	yes/no	sheep	DA	GBR	[76,77]
	footrot	yes/no	sheep	DA	GBR	[76,77]
	Interdigital dermatitis	yes/no	sheep	DA	GBR	[76,77]
	white line	yes/no	sheep	DA	GBR	[76,77]
	overgrown claws	yes/no	sheep	DA	GBR	[76,77]
	foot abscess	yes/no	sheep	DA	GBR	[76]
	granuloma	yes/no	sheep	DA	GBR	[76]
	interdigital hyperplasia	yes/no	sheep	DA	GBR	[76]
	injury	yes/no	sheep	DA	GBR	[76]
	joint infection	yes/no	sheep	DA	GBR	[76]

^1^ direct assessment: DA; recording (audio and/or video): R; sensor: S.

## References

[1] Fraser D. (2005). Animal Welfare and the Intensification of Animal Production. An Alternative Interpretation.

[2] Webster J. (1994). Animal Welfare-A Cool Eye Towards Eden.

[3] Broom D.M. (1991). Animal welfare: Concepts and measurement. J. Anim. Sci..

[4] OIE (2015). Animal Welfare and Dairy Cattle Production System. Terrestrial Animal Health Code.

[5] ISO (2016). TS 34700: Animal Welfare Management—General Requirements and Guidance for Organizations in the Food Supply Chain.

[6] EFSA (2012). Statement on the use of animal-based measures to assess the welfare of animals. EFSA J..

[7] Blokhuis H.J. (2008). International cooperation in animal welfare: The Welfare Quality® project. Acta Vet. Scand..

[8] Boyle L.A., Boyle R.M., French P. (2008). Welfare and performance of yearling dairy heifers out-wintered on a wood-chip pad or housed indoors on two levels of nutrition. Animal.

[9] Ketelaar-De Lauwere C.C., Ipema A.H., Ouwerkerk E., Hendriks M.M.W.B., Metz J.H.M., Noordhuizen J., Schouten W.G.P. (1999). Voluntary automatic milking in combination with grazing of dairy cows: Milking frequency and effects on behaviour. Appl. Anim. Behav. Sci..

[10] Leaver J.D. (1988). Management and Welfare of Animals.

[11] Loberg J., Telezhenko E., Bergsten C., Lidfors L. (2004). Behaviour and claw health in tied cows with varying access to exercise in an outdoor paddock. Appl. Anim. Behav. Sci..

[12] Hernandez-Mendo O., Von Keyserlingk M.A.G., Veira D.M., Weary D.M. (2007). Effects of pasture on lameness in dairy cows. J. Dairy Sci..

[13] Ruhland K., Gränzer W., Groth W., Pirchner F. (1999). Blood levels of hormones and metabolites, erythrocytes and leukocytes and respiration and pulse rate of heifers after alpage. J. Anim. Breed. Genet..

[14] Bertoni G., Calamari L. Animal welfare and human needs: Are they contradictory?. Proceedings of the 3rd Congress of the European Society for Agricultural and Food Ethics (Eursafe).

[15] Mattiello S. (2008). Punti critici e approccio alla valutazione del benessere nei sistemi zootecnici alpini. Congress proceedings: Benessere animale e sistemi zootecnici alpini. Quad. SoZooAlp.

[16] Conner D.S., Oppenheim D. (2008). Demand for Pasture-Raised Livestock Products: Results from Michigan Retail Surveys. J. Agribus..

[17] EFSA (2010). Application of systematic review methodology to food and feed safety assessments to support decision making. EFSA J..

[18] Higgins J.P.T., Green S. Cochrane Handbook for Systematic Reviews of Interventions Version 5.1.0, The Cochrane Collaboration. http://viawww.cochrane-handbook.org.

[19] Moher D., Liberati A., Tetzlaff J., Altman D.G. (2009). the PRISMA Group. Preferred Reporting Items for Systematic Reviews and Meta-Analyses: The PRISMA Statement. Ann. Intern. Med..

[20] Burow E., Rousing T., Thomsen P.T., Otten N.D., Sorensen J.T. (2013). Effect of grazing on the cow welfare of dairy herds evaluated by a multidimensional welfare index. Animal.

[21] Magrin L., Brscic M., Lora I., Contiero B., Cozzi G. (2016). Physiological and productive response of lactating dairy cows to the alpine transhumance at the end of the summer grazing. Ital. J. Anim. Sci..

[22] Peric T., Corazzin M., Romanzin A., Bovolenta S., Prandi A., Montillo M., Comin A. (2017). Cortisol and DHEA concentrations in the hair of dairy cows managed indoor or on pasture. Livest. Sci..

[23] Lima M.L.P., Negrão J.A., De Paz C.C.P., Grandin T. (2018). Minor corral changes and adoption of good handling practices can improve the behaviour and reduce cortisol release in Nellore cows. Trop. Anim. Health Prod..

[24] Veissier I., Van Laer E., Palme R., Moons C., Ampe B., Sonck B., Andanson S., Tuyttens F. (2018). Heat stress in cows at pasture and benefit of shade in a temperate climate region. Int. J. Biometeorol..

[25] Bovolenta S., Ventura W., Malossini F. (2002). Dairy cows grazing an alpine pasture: Effect of pattern of supplement allocation on herbage intake, body condition, milk yield and coagulation properties. Anim. Res..

[26] Hickey M.C., French P., Grant J. (2002). Out-wintering pads for finishing beef cattle: Animal production and welfare. Animal Science.

[27] Sharma A., Phillips C.J.C. (2019). Lameness in sheltered cows and its association with cow and shelter attributes. Animals.

[28] Zuliani A., Mair M., Kraševec M., Lora I., Brscic M., Cozzi G., Zupan M., Bovolenta S. (2018). A survey of selected animal-based measures of dairy cattle welfare in the Eastern Alps: Toward context-based thresholds. J. Dairy Sci..

[29] Mancera K.F., Zarza H., De Buen L.L., García A.A.C., Palacios F.M., Galindo F. (2018). Integrating links between tree coverage and cattle welfare in silvopastoral systems evaluation. Agron. Sustain. Dev..

[30] Morales A.M.T., Ceballos M.C., Londoño G.C., Cardona C.A.C., Ramírez J.F.N., Da Costa M.J.R.P. (2017). Welfare of cattle kept in intensive silvopastoral systems: A case report. Rev. Bras. Zootec..

[31] Hernandez A., Berg C., Eriksson S., Edstam L., Orihuela A., Leon H., Galina C. (2017). The welfare quality® assessment protocol: How can it be adapted to family farming dual purpose cattle raised under extensive systems in tropical conditions?. Anim. Welf..

[32] Wagner K., Brinkmann J., March S., Hinterstoißer P., Warnecke S., Schüler M., Paulsen H.M. (2018). Impact of daily grazing time on dairy cow welfare-results of the welfare quality^®^ protocol. Animals.

[33] Thompson A.J., Weary D.M., Bran J.A., Daros R.R., Hötzel M.J., Von Keyserlingk M.A.G. (2019). Lameness and lying behaviour in grazing dairy cows. J. Dairy Sci..

[34] Rice C.A., Eberhart N.L., Krawczel P.D. (2017). Prepartum lying behaviour of holstein dairy cows housed on pasture through parturition. Animals.

[35] O’Driscoll K., Lewis E., Kennedy E. (2015). Effect of feed allowance at pasture on lying behaviour and locomotory ability of dairy cows. Appl. Anim. Behav. Sci..

[36] O’Driscoll K., Lewis E., Kennedy E. (2019). Effect of feed allowance at pasture on the lying behaviour of dairy cows. Appl. Anim. Behav. Sci..

[37] Campbell D.L.M., Lea J.M., Farrer W.J., Haynes S.J., Lee C. (2017). Tech-savvy beef cattle? How heifers respond to moving virtual fence lines. Animals.

[38] Solano J., Averós X., Clemente N., Aguirre V., Estevez I., Orihuela A. (2018). Location of supplementary feed and water troughs on the sward affects movement and spatial distribution of Brahman cattle (Bos indicus). Appl. Anim. Behav. Sci..

[39] Tucker C.B., Rogers A.R., Schütz K.E. (2008). Effect of solar radiation on dairy cattle behaviour, use of shade and body temperature in a pasture-based system. Appl. Anim. Behav. Sci..

[40] Cruz E.A.D., Fischer V., Passos L.T., Da Porciuncula G.C., Stumpf M.T., Werncke D., Santos C.S. (2017). Effects of type of lesion and trimming on short-term behaviour of grazing dairy cows. Rev. Bras. Zootec..

[41] Tuomisto L., Ahola L., Martiskainen P., Kauppinen R., Huuskonen A. (2008). Comparison of time budgets of growing Hereford bulls in an uninsulated barn and in extensive forest paddocks. Livest. Sci..

[42] Blumetto O., Ruggia A., Dalmau A., Estellés F., Villagrá A. (2016). Behavioural characterisation of Holstein steers in three different production systems. Anim. Prod. Sci..

[43] Pérez-Torres L., Orihuela A., Corro M., Rubio I., Alonso M.A., Galina C.S. (2016). Effects of separation time on behavioural and physiological characteristics of Brahman cows and their calves. Appl. Anim. Behav. Sci..

[44] Corazzin M., Piasentier E., Dovier S., Bovolenta S. (2010). Effect of summer grazing on welfare of dairy cows reared in mountain tie-stall barns. Ital. J. Anim. Sci..

[45] Kohari D., Kosako T., Fukasawa M., Tsukada H. (2007). Effect of environmental enrichment by providing trees as rubbing objects in grassland: Grazing cattle need tree-grooming. Anim. Sci. J..

[46] Williams M.L., Mac Parthaláin N., Brewer P., James W.P.J., Rose M.T. (2016). A novel behavioural model of the pasture-based dairy cow from GPS data using data mining and machine learning techniques. J. Dairy Sci..

[47] Romanzin A., Corazzin M., Piasentier E., Bovolenta S. (2018). Concentrate supplement modifies the feeding behaviour of simmental cows grazing in two high mountain pastures. Animals.

[48] Lopes L.B., Eckstein C., Pina D.S., Carnevalli R.A. (2016). The influence of trees on the thermal environment and behaviour of grazing heifers in Brazilian Midwest. Trop. Anim. Health Prod..

[49] Van Laer E., Moons C.P.H., Ampe B., Sonck B., Vangeyte J., Tuyttens F. (2015). Summertime use of natural versus artificial shelter by cattle in nature reserves. Anim. Welf..

[50] Battini M., Andreoli E., Barbieri S., Mattiello S. (2011). Long-term stability of Avoidance Distance tests for on-farm assessment of dairy cow relationship to humans in alpine traditional husbandry systems. Appl. Anim. Behav. Sci..

[51] Vieyra J., Losada H., Soriano R., Cortés J., Arias L. (2000). Smallholder dairy cattle production in Xochimilco in the Southeast of Mexico City: Effect of herdsmen on spatial behaviour of cattle during restricted grazing. Livest. Res. Rural. Dev..

[52] Tresoldi G., Weary D.M., Filho L.C.P.M., Von Keyserlingk M.A.G. (2015). Social licking in pregnant dairy heifers. Animals.

[53] Boyland N.K., Mlynski D.T., James R., Brent L.J., Croft D.P. (2016). The social network structure of a dynamic group of dairy cows: From individual to group level patterns. Appl. Anim. Behav. Sci..

[54] Tuyttens F.A.M., De Graaf S., Heerkens J.L.T., Jacobs L., Nalon E., Ott S., Stadig L., Van Laer E., Ampe B. (2014). Observer bias in animal behaviour research: Can we believe what we score, if we score what we believe?. Anim. Behav..

[55] Grosso L., Battini M., Wemelsfelder F., Barbieri S., Minero M., Dalla Costa E., Mattiello S. (2016). On-farm Qualitative Behaviour Assessment of dairy goats in different housing conditions. Appl. Anim. Behav. Sci..

[56] Comin A., Prandi A., Peric T., Corazzin M., Dovier S., Bovolenta S. (2011). Hair cortisol levels in dairy cows from winter housing to summer highland grazing. Livest. Sci..

[57] Daros R.R., Hötzel M.J., Bran J.A., LeBlanc S.J., Von Keyserlingk M.A.G. (2017). Prevalence and risk factors for transition period diseases in grazing dairy cows in Brazil. Prev. Vet. Med..

[58] Bran J.A., Daros R.R., Von Keyserlingk M.A.G., LeBlanc S.J., Hötzel M.J. (2018). Cow- and herd-level factors associated with lameness in small-scale grazing dairy herds in Brazil. Prev. Vet. Med..

[59] Roche J.R., Meier S., Heiser A., Mitchell M.D., Walker C.G., Crookenden M.A., Riboni M.V., Loor J.J., Kay J.K. (2015). Effects of precalving body condition score and prepartum feeding level on production, reproduction, and health parameters in pasture-based transition dairy cows. J. Dairy Sci..

[60] Wolfger B., Timsit E., Pajor E.A., Cook N., Barkema H.W., Orsel K. (2015). Technical note: Accuracy of an ear tag-attached accelerometer to monitor rumination and feeding behaviour in feedlot cattle. J. Anim. Sci..

[61] Roche J.R., Friggens N.C., Kay J.K., Fisher M.W., Stafford K.J., Berry D.P. (2009). Body condition score and its association with dairy cow productivity, health, and welfare. J. Dairy Sci..

[62] Frey H.J., Gross J.J., Petermann R., Probst S., Bruckmaier R.M., Hofstetter P. (2018). Performance, body fat reserves and plasma metabolites in brown swiss dairy cows: Indoor feeding versus pasture-based feeding. J. Anim. Physiol. Anim. Nutr..

[63] Pryce J., Coffey M. (2001). The relationship between body condition score and reproductive performance. J. Dairy Sci..

[64] Mullins I.L., Truman C.M., Campler M.R., Bewley J.M., Costa J.H.C. (2019). Validation of a commercial automated body condition scoring system on a commercial dairy farm. Animals.

[65] Lardner H.A., Kirychuk B.D., Braul L., Willms W.D., Yarotski J. (2005). The effect of water quality on cattle performance on pasture. Aust. J. Agric. Res..

[66] Coimbra P., Machado Filho L., Nunes P., Hötzel M., De Oliveira A., Cecato U. (2010). Effect of water trough type on the drinking behaviour of pasture-based beef heifers. Animal.

[67] Bovolenta S., Ventura W., Piasentier E., Malossini F. (1998). Supplementation of dairy cows grazing an alpine pasture: Effect of concentrate level on milk production, body condition and rennet coagulation properties. Ann. Zootech..

[68] Banhazi T.M., Lehr H., Black J.L., Crabtree H., Schofield P., Tscharke M., Berckmans D. (2012). Precision Livestock Farming: An international review of scientific and commercial aspects. Int. J. Agric. Biol. Eng..

[69] Andriamandroso A.L.H., Mercatoris B., Lebeau F., Bindelle J. (2016). A review on the use of sensors to monitor cattle jaw movements and behaviour when grazing. Biotechnol. Agron. Soc. Environ..

[70] Olmos G., Boyle L., Horan B., Berry D.P., O’Connor P., Mee J.F., Hanlon A. (2009). Effect of genetic group and feed system on locomotion score, clinical lameness and hoof disorders of pasture-based Holstein-Friesian cows. Animal.

[71] Black R.A., Van Amstel S.R., Krawczel P.D. (2017). Effect of prepartum exercise, pasture turnout, or total confinement on hoof health. J. Dairy Sci..

[72] Ranjbar S., Rabiee A.R., Gunn A., House J.K. (2016). Identifying risk factors associated with lameness in pasture-based dairy herds. J. Dairy Sci..

[73] Laven R.A., Fabian J. (2016). Applying animal-based welfare assessments on New Zealand dairy farms: Feasibility and a comparison with United Kingdom data. NZ Vet. J..

[74] Welfare Quality (2009). Welfare Quality Assessment Protocol for Cattle.

[75] Burow E., Thomsen P.T., Rousing T., Sørensen J.T. (2013). Daily grazing time as a risk factor for alterations at the hock joint integument in dairy cows. Animal.

[76] Angell J.W., Grove-White D.H., Duncan J.S. (2015). Sheep and farm level factors associated with contagious ovine digital dermatitis: A longitudinal repeated cross-sectional study of sheep on six farms. Prev. Vet. Med..

[77] Angell J.W., Grove-White D.H., Duncan J.S. (2018). Sheep and farm level factors associated with footrot: A longitudinal repeated cross-sectional study of sheep on six farms in the UK. Vet. Rec..

[78] Phythian C.J., Michalopoulou E., Cripps P.J., Duncan J.S., Wemelsfelder F. (2016). On-farm qualitative behaviour assessment in sheep: Repeated measurements across time, and association with physical indicators of flock health and welfare. Appl. Anim. Behav. Sci..

[79] Munoz C., Campbell A., Barber S., Hemsworth P., Doyle R. (2018). Using longitudinal assessment on extensively managed ewes to quantify welfare compromise and risks. Animals.

[80] McLennan K., Skillings E.A., Rebelo C.J., Corke M.J., Moreira M.A., Morton A.J., Constantino-Casas F. (2015). Technical note: Validation of an automatic recording system to assess behavioural activity level in sheep (Ovis aries). Small Rumin. Res..

[81] Di Virgilio A., Morales J.M., Lambertucci S.A., Shepard E.L.C., Wilson R.P. (2018). Multi-dimensional Precision Livestock Farming: A potential toolbox for sustainable rangeland management. PeerJ..

[82] Munoz C., Campbell A., Hemsworth P., Doyle R. (2018). Animal-based measures to assess the welfare of extensively managed ewes. Animals.

[83] Sarout B.N., Waterhouse A., Duthie C., Poli C.H., Haskell M.J., Berger A., Umstatter C. (2018). Assessment of circadian rhythm of activity combined with random regression model as a novel approach to monitoring sheep in an extensive system. Appl. Anim. Behav. Sci..

[84] McGregor B.A., Butler K.L. (2008). Relationship of body condition score, live weight, stocking rate and grazing system to the mortality of Angora goats from hypothermia and their use in the assessment of welfare risks. Aust. Vet. J..

[85] Scocco P., Piermarteri K., Malfatti A., Tardella F.M., Catorci A. (2016). Effects of summer rainfall variations on sheep body state and farming sustainability in sub-Mediterranean pastoral systems. Span. J. Agric. Res..

[86] Scocco P., Piermarteri K., Malfatti A., Tardella F.M., Catorci A. (2016). Increase of drought stress negatively affects the sustainability of extensive sheep farming in sub-Mediterranean climate. J. Arid Environ..

[87] Morgan-Davies C., Waterhouse A., Pollock M.L., Milner J.M. (2008). Body condition score as an indicator of ewe survival under extensive conditions. Anim. Welf..

[88] González-García E., Alhamada M., Pradel J., Douls S., Parisot S., Bocquier F., Menassol J.B., Llach I., González L.A. (2018). A mobile and automated walk-over-weighing system for a close and remote monitoring of liveweight in sheep. Comput. Electron. Agric..

[89] Mattiello S., Battini M., De Rosa G., Napolitano F., Dwyer C. (2019). How can we assess positive welfare in ruminants?. Animals.

